# Development of image quality related reference doses called acceptable quality doses (AQD) in paediatric CT exams in Qatar

**DOI:** 10.1007/s00330-020-07375-7

**Published:** 2020-11-11

**Authors:** Mohammad Hassan Kharita, Huda AlNaemi, Vishwanatha Kini, Shady Alkhazzam, Madan M. Rehani

**Affiliations:** 1 Hamad Medical Corporation, Occupational Health and Safety, Radiation Safety Section, Doha, Qatar; 2 Massachusetts General Hospital (MGH), Boston, USA

**Keywords:** Tomography, X-ray computed, Abdomen, Head, Radiologist

## Abstract

**Objectives:**

To describe first experience of integrating assessment of image quality in paediatric X-ray computed tomography (CT) with analysis of the radiation dose indices to develop reference doses called acceptable quality dose (AQD).

**Methods:**

Image quality was scored by the radiologists at a tertiary care hospital in Qatar on a scale of 0 to 4 using the recently published scoring criteria. The patients undergoing head, chest and abdomen CT were divided in different weight groups as follows: < 5 kg, 5–< 15 kg, 15–< 30 kg, 30–< 50 kg, 50–< 80 kg and > 80 kg. The images that were clinically acceptable (score of 3) were included for assessment of median values of CTDIvol and DLP to obtain AQDs in different weight groups.

**Results:**

After initial training in image quality scoring of CT images of 49 patients by three radiologists, the study on 715 patients indicated 665 studies (93%) were clinically acceptable as per scoring criteria. The median CTDI_vol_ values for the above weight groups were 16, 20, 22, 22, 27 and 27 mGy and the median DLP values for these weight groups were 271, 377, 463, 486, 568 and 570 mGy cm, respectively, for head CT. Similar values are presented for chest and abdomen CTs.

**Conclusions:**

The first ever experience of starting with image quality assessment and integrating it with analysis of dose indices to obtain AQD values shall provide a workable model for others and values for comparison within the facility and in other facilities leading to optimisation.

**Key Points:**

• *The first study to integrate image quality assessment with analysis of patient dose indices shows feasibility for routine practice in other centres.*

• *The values of acceptable quality dose (AQD) were provided for head, chest and abdomen CT of children divided into weight groups rather than age. They shall act as reference values for future studies.*

• *Verification of our findings on proportional increase in exposure parameters (CTDIvol and DLP) with weight by other investigators shall be helpful.*

## Introduction

Some tissues in children are more radiosensitive than in adults and the need for higher concern in radiation protection of children has been recognised [1, 2]. Computed tomography (CT) in children is a well-established imaging modality but concerns on radiation risks have frequently been raised both in professional circles and in public media [3–9]. Reducing radiation exposure to children undergoing CT exams while maintaining diagnostic information has repeatedly been emphasised [2, 3, 5]. Since there are no dose limits for patients, unlike the occupational workers, the concept of diagnostic reference level (DRL) was propagated by ICRP in 1996 [10] and adopted in regulatory framework of many countries. While DRLs have proved to be of value for the purpose for which they were developed, that is, cutting down exposures higher than the 75th percentile of dose distribution, the limitations of DRLs in overall scheme of optimisation have increasingly been identified [11, 12]. Some recent papers provide detailed analysis of long-term experience of France in DRLs and describe the good, the bad and the ugly aspects of DRLs [13–15]. The bad aspects of DRL include the following facts: First, the tendency to view DRLs as a “speed limit” and leading one to believe that being below the DRL means optimisation has been achieved. Second, DRLs are defined for standard-sized adult patients only. Third, definition of DRLs requires many years of data collection and legal and administrative process during which technology normally would have changed [11–13]. The ugly aspects of DRLs include the following facts: First, DRLs are not applicable to individual patients. Second, DRLs are not available for many clinical indications. Third, image quality has been neglected so far when estimating DRLs. Fourth, DRLs may be occasionally perceived as a dose limit [11–13]. In view of these limitations, alternative approaches are most urgently needed.

The concept of acceptable quality dose (AQD) was introduced to attend to many limitations of DRL [11]. This concept requires first assessing the image quality by the radiologist and then analyse dose indices only of images that are considered acceptable. Thus, image quality is recommended to be starting point rather than dose. Furthermore, the image quality scoring criteria (IQSC) for paediatric CT have been provided [16]. There is lack of publication on this approach of AQD. The purpose of the current study is to provide our experience with image quality assessment using the IQSC and provide assessment of AQD for which data is not yet available from other sources, being a new concept. The work was done in Qatar.

## Materials and methods

The data was collected in the Hamad General Hospital (HGH) in Qatar on four CT scanners (one Siemens Somatom Definition Flash 128 slices, two Siemens Somatom Sensation 64 slice and one Philips Brilliance iCT 256 slices) in the Clinical Imaging Department.

This retrospective study consists of two phases, as described below. In both phases, three groups of CT examinations (based on anatomical regions) of head, chest and abdomen were selected (for the time being, only single-phase cases without contrast were considered). These examinations were the most common in the four CT rooms participating in the study from HGH. The iterative image reconstruction algorithms (SAFIRE, VEO) were regularly applied and imaging performed using CARE dose in helical mode.

### Image quality scoring (phase I)

Phase I of the study consisted of subjective assessment of image quality score using IQSC [16]. Readers are referred to [16] for more details, but in brief, the scoring scale was 0 to 4, as below: Score 0s = Desired features not seen; 0i = Anatomy not included in the images; 1 = Unacceptable quality (images do not allow diagnostic interpretation); 2 = Limited quality (images are adequate only for limited clinical interpretation due to high noise); 3 = Adequate quality (images are just adequate for diagnostic interpretation); and 4 = Higher than needed quality (images are much better than needed for interpretation: images with very little noise). For detailed criteria for different body parts and indications, please refer to publication [16]. Essentially, this is like Likert scale.

In this phase, three paediatric radiologists (with 15, 20 and 25 years of experience) participated in independent evaluation of 49 randomly selected CT examinations that included head (17 patients), chest (20) and abdomen (12 patients). PACS monitor allowed manipulation of the window settings of the images for optimal viewing as desired by the individual radiologist. Radiologists could change the window width and levels as per their preferences. This helped us to test the interobserver variability and also helped radiologist to get trained on image quality scoring.

### AQD estimation (phase II)

After having assessed interobserver variability in phase I, in phase II, the assessment of image quality score by any radiologist in paediatric division with experience 15–25 years was accepted for radiation dose analysis part of AQD estimation. The radiologists were asked to assign image quality score based on IQSC [16]. At the end of each day, the excel sheets with scores were consolidated by one person assigned to this project and this was done for all days of the data collection period. Only those imaging studies with score of exactly 3 were then used for analysis of doses as below. In this approach, the images that were associated with higher than needed image quality (score 4), or not acceptable quality images (score 2 or less), were excluded for AQD assessment. The purpose of this was to detect imaging studies of acceptable quality.

In phase II, data were collected retrospectively in CT studies of 715 children under the age of 15 that were grouped into six weight groups as provided by a recent European Commission project [17]. These were < 5 kg (neonates), 5–< 15 kg (infants, toddler and early childhood), 15–< 30 kg (middle childhood), 30–< 50 kg (early adolescence), 50–< 80 kg (late adolescence) and > 80 kg (obese). The dose data were collected with additional information which included the region of examination, the patient-specific data (sex, age and weight), dose indices CTDI_vol_ and DLP and image quality score. The median values for CTDI_vol_ and DLP were determined for each weight category and that becomes AQD for corresponding weight group.

## Results

The results of image quality scoring for 49 patients in phase I are presented in Tables 1 and 2.

**Table 1:** Median of image quality scores using IQSC for three common CT protocols

	Reader 1	Reader 2	Reader 3	Median
Head (17 patients)	3	3	3	3
Chest (20 patients)	3	3	3	3
Abdomen (12 patients)	3	3	3	3

**Table 2:** Frequency of subjective image quality score (1–4) for the three radiologists

Region/body part	Score	Radiologist 1	Radiologist 2	Radiologist 3
Head (17 patients)	1	0 (0%)	0 (0%)	0 (0%)
	2	6 (35%)	8 (47%)	8 (47%)
	3	11 (65%)	9 (53%)	9 (53%)
	4	0 (0%)	0 (0%)	0 (0%)
Chest (20 patients)	1	0 (0%)	0 (0%)	0 (0%)
	2	0 (0%)	1 (5%)	1 (5%)
	3	20 (100%)	19 (95%)	18 (90%)
	4	0 (0%)	0 (0%)	1 (5%)
Abdomen (12 patients)	1	0 (0%)	0 (0%)	0 (0%)
	2	0 (0%)	0 (0%)	2 (17%)
	3	12 (100%)	12 (100%)	9 (75%)
	4	0 (0%)	0 (0%)	1 (8%)

The median image quality scores for paediatric CT exams of head, chest and abdomen for each of the three readers were the score of 3 for all 49 patients. The score given by three radiologists was 3 (in 81% of all CT exams), score of 4 for 1% of CT exams and score of 2 for 18% exams. The kappa statistics were used to assess the interobserver variability. The interobserver agreement among the three readers (acceptable image quality [scores 3 or 4] vs sub-optimal image quality ([scores 1 and 2]) was good to very good (kappa 0.74–0.89).

In keeping with the concept of AQD, where images of acceptable image quality are to be included for dose analysis, it was found in phase II study that CT images of 50 out of 715 patients (nearly 7%) had a score differing from a score of 3 (40 patients with score of 2 and 10 patients with score 4) and thus they were not included in AQD estimation. As a result, only CT exams of 665 patients with an acceptable level of image quality (score 3) were classified into head, chest and abdomen CTs and the numbers were 353, 111 and 201 respectively. Head CT forms the largest component (53%) followed by abdomen 30% and chest 17%. Tables 3, 4 and 5 provide further distributions in different weight groups and corresponding CTDI_vol_, DLP and AQDs.

**Table 3:** Distribution of patients in different weight groups for 353 head CT examinations, CTDI_vol_ and DLP per weight group (AQD)

	Region of exam	Head					
	Weight group	< 5 kg	5–< 15 kg	15–< 30 kg	30–< 50 kg	50–< 80 kg	> 80 kg
	Number of patients	11	101	50	70	88	33
	Avg. age (year)	0.5	3.1	6.6	8.4	11.9	11.9
CTDI_vol_ mGy	Median	16	20	22	22	27	27
	75th%	20	21	27	27	28	28
	Mean ± SD	15 ± 8	20 ± 5	25 ± 10	25 ± 9	30 ± 12	29 ± 9
DLP mGy cm	Median	271	377	463	486	568	570
	75th%	338	451	615	572	689	622
	Mean ± SD	252 ± 153	400 ± 128	514 ± 216	511 ± 154	649 ± 301	615 ± 219

**Table 4:** Distribution of patients in different weight groups for 111 chest CT examinations, CTDI_vol_ and DLP per weight group (AQD) and European DRL

	Region of exam	Chest					
	Weight group	< 5 kg	5–< 15 kg	15–< 30 kg	30–< 50 kg	50–< 80 kg	> 80 kg
	Number of patients	20	29	24	29	9	
	Avg. age (year)	0.5	2.4	7.2	11.0	12.1	
CTDI_vol_ mGy	Median	1	2	2	3	4	
	75th%	1	2	3	4	5	
	Mean ± SD	1 ± 0	2 ± 1	2 ± 1	4 ± 3	5 ± 3	
DLP mGy cm	Median	12	33	59	93	109	
	75th%	14	53	113	145	177	
	Mean ± SD	13 ± 5	48 ± 44	86 ± 73	140 ± 139	201 ± 178	
	European DRL 2018	35	50	70	115	200

**Table 5:** Distribution of patients in different weight groups for 201 abdomen CT examinations, CTDI_vol_ and DLP per weight group (AQD) and European DRL

	Region of exam	Abdomen					
	Weight group	< 5 kg	5–< 15 kg	15–< 30 kg	30–< 50 kg	50–< 80 kg	> 80 kg
	Number of patients	4	32	35	45	62	23
	Avg. age (year)	0.5	2.0	7.1	12.3	13.8	14.0
CTDI_vol_ mGy	Median	2	3	3	6	7	11
	75th%	3	3	5	7	9	15
	Mean ± SD	3 ± 2	3 ± 1	4 ± 2	6 ± 3	8 ± 3	11 ± 5
DLP mGy cm	Median	54	75	122	260	377	605
	75th%	102	98	196	331	474	776
	Mean ± SD	89 ± 81	95 ± 65	138 ± 75	333 ± 258	390 ± 168	662 ± 407
	European DRL 2018	45	120	150	210	480	

It is interesting to note that the percentages of children in the weight group 50–< 80 and > 80 kg are not small as it forms 34% of head, 8% of chest and 42% of abdomen CT exams and nearly 32% of the total. This is a sizable fraction and their weight is similar to adults which further highlights importance of weight or body build classification, rather than age.

**Fig. 1:**
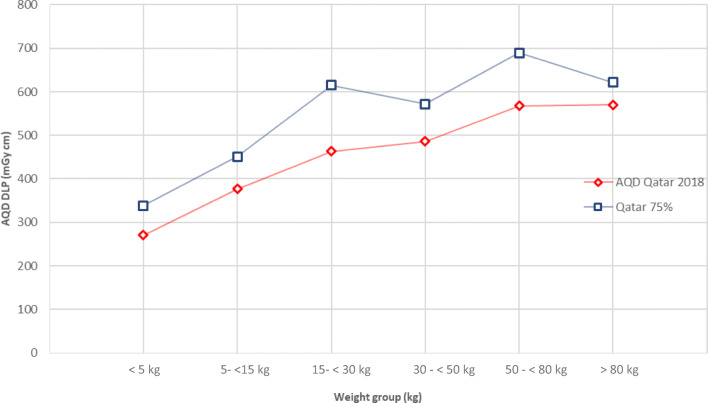
AQD and 75th % of Qatar DLP for paediatric head CT examinations

For head CT, the distribution of 353 patients in weight groups is 3% neonates, 29% infants, 14% middle childhood, 20% early adolescence, 25% late adolescence and 9% obese children. The median CTDI_vol_ values for these groups were 16, 20, 22, 22, 27 and 27 mGy, respectively. The median DLP values for these weight groups were 271, 377, 463, 486, 568 and 570 mGy cm, respectively. As can be seen from Fig. 1, the dose values reach a plateau at 50 kg but they show a continued increase at lower weights.

Notwithstanding the fact that the head size is known to almost reach the adult size by the age of 2 years or latest 5 years, Table 3 presents data of 353 CT head examinations distributed in 6 weight groups, providing average age for each group, and median, mean, standard deviation for CTDI_vol_ and DLP. The median values of CTDI_vol_ and DLP become AQDs for the respective group.

For chest CT, the distribution of 111 patients in different weight groups is as follows: 18% neonates, 26% infants, 22% middle childhood, 26% early adolescence and 8% late adolescence children. The median CTDI_vol_ values for these groups were 1, 2, 2, 3 and 4 mGy, respectively. The median values of DLP for these weight groups were 12, 33, 59, 93 and 109 mGy cm, respectively.

For abdomen CT, the distribution of 201 patients in different weight groups is as follows: 2% neonates, 16% infants, 17% mid-childhood, 22% early adolescence, 31% late adolescence and 11% obese children. The median CTDI_vol_ values for these groups were 2, 3, 3, 6, 7 and 11 mGy, respectively. The median values of DLP for these weight groups were 54, 75, 122, 260, 377 and 605 mGy cm, respectively.

Tables 4 and 5 present data for CT chest and abdomen exam, respectively, along with median values for each weight group which becomes respective AQD.

**Fig. 2:**
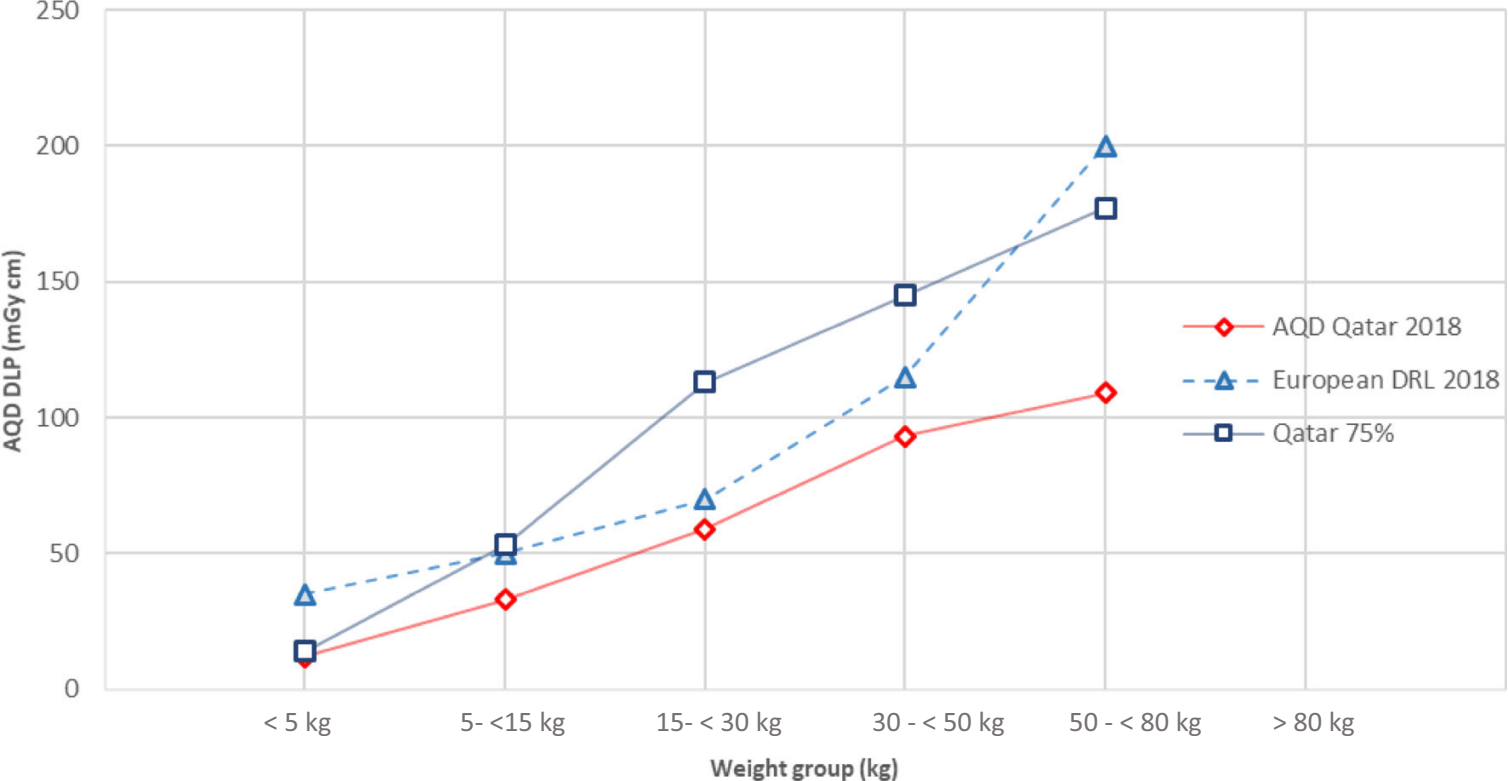
AQD, 75th % of Qatar DLP and European DRL study for paediatric chest CT examinations

**Fig. 3:**
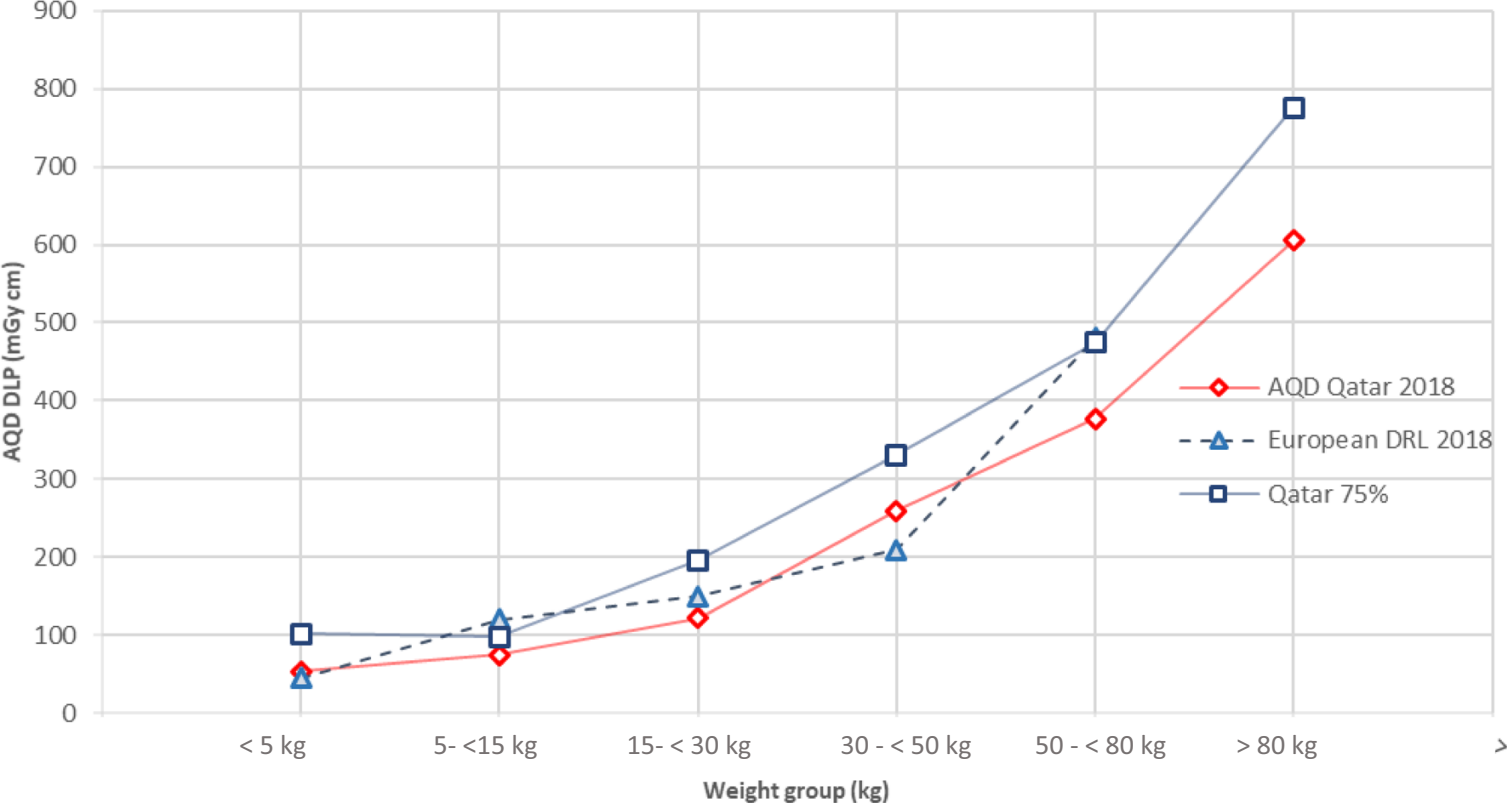
AQD, 75th % of Qatar DLP and European DRL study for paediatric abdomen CT examinations

Overall, the AQD results are presented in Figs.1, 2 and 3 for head, chest and abdomen respectively. In the backdrop of knowledge that the head size does not change significantly after the age of 2 or 5 years, the pattern of curve in Fig. 1 indicating nearly proportional increase with weight provides interesting observations which should be explored by others.

Since the median values for European or US data are not available for the new weight groups proposed by EC [17] in recent years, the AQD values cannot be compared with others. However, for comparison purpose, we have also provided our 75th percentile values in tables and figures. In Figs. 2 and 3, comparison of 75th percentile of this study with European DRLs (2018) [17] is presented. The pattern in Fig. 2 shows that our 75th % values for chest CT are in line with European DRLs except for weight group 15–< 30 kg and 30–< 50 kg where they are much higher. A similar observation can be made from Fig. 3 for abdomen CT where our values are in line with the European DRLs except for weight group 15–< 30 kg and 30–< 50 kg where our values are higher than European DRLs.

## Discussion

The first study to provide AQDs for paediatric CT patients is presented here. Even though Qatar is a small country, there are no DRLs established for CT exams. In fact, most countries in the region lack DRLs. AQD was proposed as a ground-level-based approach rather than DRLs that use top-down approach. AQD starts with image quality assessment and utilises only images of acceptable quality for dose analysis. There are interesting findings in this study. Firstly, 50 out of 715 (nearly 7%) of CT exams were considered to be not clinically acceptable by the radiologists using the scoring criteria. This is not an ignorable fraction. That implies that in most dose surveys where image quality is not assessed and not documented, there is a chance that reasonable level of exams that are not clinically acceptable are also counted and the dose indices presented will not provide true values of clinically acceptable exams. Secondly, the usual approach in many dose studies in children has taken age classification with not so many using weight classification [18]. Our study demonstrates that the fraction of the children (up to age 15) but with weight 50 kg and above is not small in Qatar as they form 32%. This is a sizable fraction and their weight is similar to adults. Moreover, children with > 80 kg were found not only in higher age bracket but also in lower ages (Tables 3 and 5). A look at the literature does provide evidence that childhood obesity is a growing healthcare epidemic in Qatar [19]. As per this report, 28.7% of children have overweight among boys, and 18.8% among girls in Qatar. This further highlights importance of weight or body build classification, rather than age. We did not have direct automatic access to equivalent body diameter or water equivalent diameter or size-specific dose estimates (SSDE) which is the case in most countries and thus weight is still the most convenient parameter till such time electronic estimates of body build are easily available and they can be used in the concept of AQD. Manual assessment of diameters can be fraught with inaccuracies and variations with operator.

**Fig. 4:**
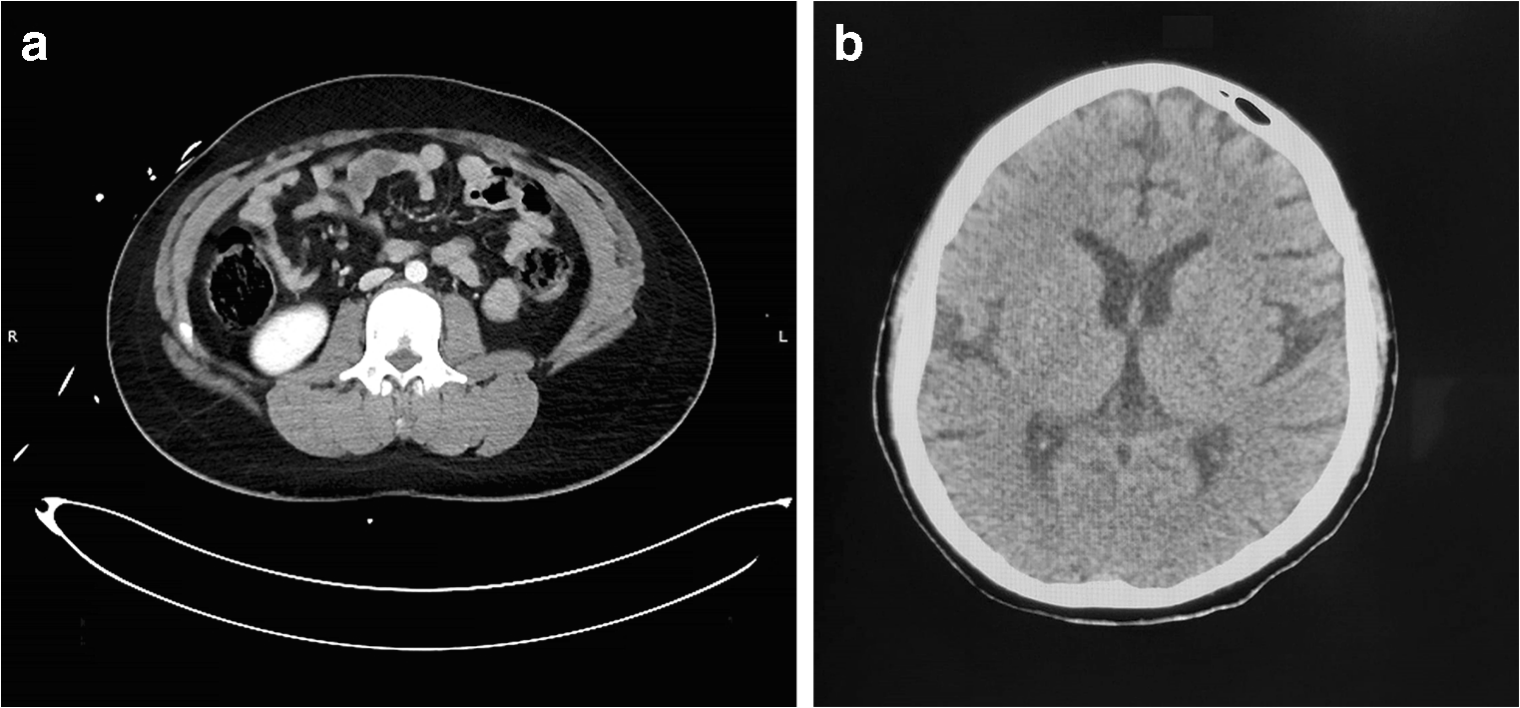
**a** Presents image from the study with score of 3. **b** Presents image from the study with score of 4

It should be understood that the score of 4 depends upon training and orientation of the readers (radiologists). In good centres, where awareness is created about acceptance of images with some noise rather than crisp images, the radiologists can discern images of higher than necessary quality [11, 16]. Thus, the score of 4 may be assigned more often by dose conscious reader than others. Awareness about what features should be visible and to what extent helps in this and that is where the criteria becomes important [16]. Figure 4a presents images of a study with score of 3, whereas Fig. 4b present image of another patient with score of 4 indicating that some degree on noise in Fig. 4a does not interfere with diagnostic interpretation, but the image does appear grainy.

Our observations on increase in dose indices for head CT with patient’s weight need verification by others.

There are many studies in the world that have provided DRLs for paediatric CT, such as Italian nationwide survey (2015) [20], Australia (2016) [21], Australia (2018) [22], France (2011) [23], France (2020) [14, 15], Japan (2015) [24], Germany (2016) [25], England (2018) [26], Finland (2015) [27], the results from multicentre study in California, USA (2015) [28] and the European DRL (2018) [17].

We believe that the concept of AQD that integrates image quality with dose shall be found to be meaningful and our results shall provide reference values for future studies on AQD. Image quality has long been left unattended, even though it has always been mentioned. The scoring criteria [16] in this respect and our positive experience should provide motivating situation. The approach uses power of the facility and does not depend upon concerted actions driven by national bodies.

### Challenges and limitations of the study

The study was conducted in a busy hospital where clinical orientation rather than research dominates day-to-day activities of radiological professionals. It is much easier to perform dose analysis which most studies do. But assessment of image quality by the radiologist and integration with dose assessment provides its own challenges. Non-availability of a diagnostic medical physicist in radiology department provided further challenges. With that background and keeping in mind that there is no report available, whereas similar studies have been done, this work should be seen as initial attempt whereby refinements with time can be assumed to happen. These pertain to collection of data on indication-based CT exams rather than body part/region based, using other measures of body build than weight and having larger sample than included in this study. It is hoped that other studies will provide AQDs on more exams that will provide opportunity to compare results and achieve better optimisation.

## Conclusions

The concept of AQD has a number of inherent advantages, namely it starts with a facility rather than national levels and thus promotes facility-based actions; is based on clinically acceptable image quality that is the primary goal of any imaging rather than the dose that is the secondary parameter; covers all three crucial parameters, namely image quality, dose and the patient’s body build; and views optimisation truly from the angle of optimisation rather than just exclusion of outliers.

## Abbreviations

AQD: Acceptable quality dose
CT: Computed tomography
CTDI_vol_: Volume CT dose index
DLP: Dose-length product
DRLs: Diagnostic reference levels
HGH: Hamad General Hospital
ICRP: International Commission on Radiological Protection
IQSC: Image Quality Scoring Criteria
PACS: Picture archiving and communication system
